# The Association between Noise, Cortisol and Heart Rate in a Small-Scale Gold Mining Community—A Pilot Study

**DOI:** 10.3390/ijerph120809952

**Published:** 2015-08-21

**Authors:** Allyson Green, Andrew D. Jones, Kan Sun, Richard L. Neitzel

**Affiliations:** 1 Department of Environmental Health Sciences, University of Michigan School of Public Health, Ann Arbor, MI 48109, USA; E-Mails: aggreen@umich.edu (A.G.); jonesand@umich.edu (A.D.J.); kansun@umich.edu (K.S.); 2 Risk Science Center, University of Michigan School of Public Health, Ann Arbor, MI 48109, USA

**Keywords:** small-scale gold mining, ASGM, dietary diversity, salivary cortisol, stress, heart rate, noise exposure, Ghana, health determinants

## Abstract

We performed a cross-sectional pilot study on salivary cortisol, heart rate, and personal noise exposures in a small-scale gold mining village in northeastern Ghana in 2013. Cortisol level changes between morning and evening among participants showed a relatively low decline in cortisol through the day (−1.44 ± 4.27 nmol/L, n = 18), a pattern consistent with chronic stress. A multiple linear regression, adjusting for age, sex, smoking status, and time between samples indicated a significant increase of 0.25 nmol/L cortisol from afternoon to evening per 1 dBA increase in equivalent continuous noise exposure (L_eq_) over that period (95% CI: 0.08–0.42, Adj R^2^ = 0.502, n = 17). A mixed effect linear regression model adjusting for age and sex indicated a significant increase of 0.29 heart beats per minute (BPM) for every 1 dB increase in L_eq_. Using standard deviations (SDs) as measures of variation, and adjusting for age and sex over the sampling period, we found that a 1 dBA increase in noise variation over time (L_eq_ SD) was associated with a 0.5 BPM increase in heart rate SD (95% CI: 0.04–−0.9, Adj. R^2^ = 0.229, n = 16). Noise levels were consistently high, with 24-hour average L_eq_ exposures ranging from 56.9 to 92.0 dBA, with a mean daily L_eq_ of 82.2 ± 7.3 dBA (mean monitoring duration 22.1 ± 1.9 hours, n = 22). Ninety-five percent of participants had 24-hour average L_eq_ noise levels over the 70 dBA World health Organization (WHO) guideline level for prevention of hearing loss. These findings suggest that small-scale mining communities may face multiple, potentially additive health risks that are not yet well documented, including hearing loss and cardiovascular effects of stress and noise.

## 1. Introduction

Artisanal and small-scale gold mining (ASGM) communities worldwide face a number of public health concerns, including direct and indirect exposure to hazardous materials present in gold ore and those used in amalgamation (e.g., mercury); degraded environmental quality through water pollution and deforestation; and dust and noise exposures resulting from the mining process [1,2,3]. As a key producer in the global gold market, Ghana relies on gold for almost 40% of its total exports, with production having risen 700% since 1980 [4]. Much of that increase has come from small-scale gold mines, which now provide an estimated 20–30% of total gold output worldwide [5]. Mining activities are often interspersed with residential and commercial areas, resulting in comingled occupational and community exposures. Social stressors such as migration and economic hierarchies have also been documented in ASGM communities [6], which are often rural and impoverished with limited healthcare and sanitation [7].

While many potential stressors affect mining community members, studies focusing on stress resulting from mining are rare [8,9,10]. Chronic psychosocial stress from sources such as work, home life, and socioeconomic status, has been associated with cardiovascular disease [11,12], acute myocardial infarction [13], inflammation [14], hypertension [15], and immune dysregulation [16]. The endocrine response in the hypothalamic-pituitary-adrenocorticol axis (HPA) is one of the physiological mediators of disease linked with psychological stress, with cortisol in saliva increasingly used as a hormonal biomarker for stress in research. Generally high average cortisol levels and a relatively low drop in cortisol levels from morning to evening are seen with chronic stressors [17].

The mining process involves multiple steps with the potential for elevated occupational and community noise levels, but noise exposures associated with ASGM activities have not been studied previously. Noise exposure is associated with hearing impairment, hypertension, ischemic heart disease, annoyance, degraded school performance, and sleep disturbance [18,19]. Noise is also increasingly being linked to stress in studies using both self-reported measures of psychological stress and physiological indicators (e.g., cortisol and heart rate variability) [20,21], though a recent review noted that the effects of noise on stress and cortisol require additional study [22].

The goal of this pilot study was to document noise levels and evaluate the relationship between noise and stress indicators in Kejetia, a mining community in the Talensi District in the Upper East Region of Ghana.

## 2. Experimental Section

### 2.1. Overview

This study documented patterns in stress, and noise exposure through qualitative and quantitative measures administered to participants in Kejetia in 2013. Surveys explored perceived stress and perception of noise in the community. Participants gave saliva samples and wore heart rate monitors and dosimeters to monitor changes in stress biomarkers and noise exposure over a 24-hour period. These data were matched in time in order to investigate patterns according to confounding factors such as age and sex.

### 2.2. Site Description and Interview Process

The small-scale gold-mining town of Kejetia in the Talensi District was founded in 1995 and grew to around 15,000 by 2000. Ten years later, Kejetia was home to an estimated 2500 people, illustrating the unstable and transient nature of communities built around ASGM activities [23]. Non-mining residents may work as food or water vendors, mechanics, or other service providers, but ASGM activity is prolific throughout the community.

Interviews and sample collection took place at participants’ homes or workplaces in Kejetia. All surveys and instruments were delivered in the participant’s preferred language through local translators as described in Long *et al*., this issue [24]. Stress and noise data were collected in April 2013 from a convenience sample of participants who participated in a previous study. Participants were originally chosen from clusters (as described in Long *et al*., this issue [24]), and the convenience sample included the first few people found from the original clusters who were willing and able to participate. All subjects gave their informed consent to participate in the study. The University of Michigan Institutional Review Board (HUM00028444 and HUM00073615) approved the study’s involvement of adult human subjects. Subjects were compensated for their participation with cash approximately equal to what they would have normally earned during the several hours that they spent participating in the study.

### 2.3. Stress and Noise

Perceived stress was measured during an interview using five items from the 14-item Perceived Stress Scale (PSS) [25]. Using the original PSS wording of the questions (translated during the interviews) participants were asked to report how often in the last month (“never”, “almost never”, “sometimes”, “fairly often”, or “very often”) they had felt upset, unable to control important things in their life, nervous or stressed, confident, and angered. These particular PSS questions were chosen, instead of the typical PSS4 questions, in consultation with translators because they were thought to be more easily translatable than others. While the PSS has not been validated in this context, it was developed for general use and has been used previously in Eastern Ghana [26].

Physiological stress and personal noise exposures were measured during the 24 hour period after the interview, with total duration of measurement ranging from 17–24 hours, depending on interview schedules and participant availability. Verbal and pictorial instructions reminded participants to avoid eating any food or drinking dairy products within an hour of each sample, to abstain from alcohol consumption, and to rinse their mouth out with water 10 minutes before each sample. Equipment and saliva samples were collected the following day, and participants were asked to recall the type, approximate start time, and duration of all activities they participated in during the sample period. Participants gave a saliva sample with Salimetrics Oral Swabs (Salimetrics Europe, Ltd.) held under the tongue for 60 seconds at the time of the interview (afternoon sample), before going to bed (evening sample), and immediately upon waking the next day (morning sample). Times for each sample were recorded. Samples were kept unrefrigerated and processed within three weeks at the University of Michigan Core Assay Facility using a DPC Coat-A-Count Cortisol modified protocol for saliva. The diurnal change in salivary cortisol level was calculated by subtracting the morning sample value from the evening value so that a negative change between samples corresponded to a decrease in cortisol over time. This morning to evening change measurement differs from standard practice in the literature since the evening sample preceded the morning sample, but the sampling procedure was necessitated by constraints on interactions with subjects. We assumed consistency in variables such as sleep duration and awakening time, which have been identified as potential confounders affecting salivary cortisol levels [27].

Heart rate (HR, in beats per minute, or bpm) was logged in 5 second intervals with a Garmin FR70 Fitness Watch with Heart Rate Monitor (Garmin, Olathe, KS, USA) worn by participants. Personal noise exposure was measured using an Etymotic Research Inc. ER-200D Personal Noise Dosimeter (Etymotic Research, Elk Grove Village, IL, USA) attached to the shirt collar, logging equivalent continuous sound pressure levels every 3.75 minutes. An L_eq_ measurement provides a standardized measure of the average sound pressure level for both continuous and time-varying noise. The dosimeter approximated the performance of a Type 2 dosimeter [28] (exchange rate 3 dB, threshold 70 dBA, criterion level 85 dBA) and had a measurement range of 70–130 dBA. The dosimeters datalogged noise levels of 0 dBA for intervals in which noise levels never exceeded 70 dBA (e.g., 70 dBA limit of detection). All datapoints with L_eq_ = 0 were recoded as 70/$\sqrt{2}$ to better estimate the likely distribution of noise levels below 70 dBA [29]. Dosimeter timepoints beyond 24 hours were excluded. A daily average L_eq_ was calculated for each participant over 24 hours or the duration of time the dosimeter was worn, whichever was greater. Within that timeframe, a separate average L_eq_ for certain activities was also calculated based on the timing of activities reported by subjects when turning in their dosimeters. To match noise exposures with salivary cortisol responses, average L_eq_ for the time between samples was also calculated for each participant.

HR data were synchronized in time with sound level measurements, and the 5 second HR datapoints were averaged to match the 3.75 min intervals of the dosimeters. Mean and standard deviation (SD) HR and L_eq_ were calculated for the duration of each reported activity, but these HR-paired L_eq_ values differed in some cases from those calculated solely on dosimeter data because HR data were often missing due to HR monitor connectivity issues.

### 2.4. Analysis

All statistics were performed using IBM SPSS Statistics 21.0. Descriptive statistics were computed for all variables, and Shapiro-Wilk tests were used to determine distributions and appropriateness of tests. No variables were transformed, as the assumptions for inferential testing via regression were not violated. Exceedance fractions (*i.e.*, the fraction of exposures above the recommended 85 dBA noise exposure limit) were computed for the dosimetry L_eq_ data. Differences between men and women, miners and nonminers, and education levels were examined for changes in cortisol and L_eq_ (Mann-Whitney U, Kruskall-Wallace tests). Differences in HR and L_eq_ across activities were compared with Wilcoxon Signed Rank tests. Pearson or Spearman correlations were calculated for variables of interest, and Cronbach’s alpha was calculated for the set of personal concerns and PSS questions.

The diurnal change in cortisol level was used as the outcome in backward stepwise linear regression, with *p* = 0.05 as a threshold for entry into the model and *p* = 0.1 for removal. In addition to predictors identified as significant in stepwise regression, other predictors were included in the final adjusted models, regardless of coefficient significance, based on literature review and significant relationships to the outcome. This method was also followed to create a model for variation in HR using the standard deviation of HR and L_eq_. Finally, a mixed effects linear model including a random effect for participant ID was used to evaluate the association between HR and L_eq_. The average HR over a 3.75 minute interval was used as the outcome. After adjusting for age and sex, the fixed effect of L_eq_ was estimated.

## 3. Results

### 3.1. Demographics

Twenty-two individuals from 16 households participated in the study (Table 1). More than 50% of subjects reported less than a primary school education and were currently working as miners.

**Table 1:** Subject demographics (N = 22).

		Total			Female			Male	
		Mean	SD		Mean	SD		Mean	SD
Average Age (years)		**34.1**	**10.3**		32.5	9.8		36.0	11.1
Years Lived in Kejetia		**10.5**	**6.7**		10.1	6.9		11.0	6.7
							
		**N**	**%**		**n**	**%**		**n**	**%**
Gender		**22**	**-**		12	55%		10	45%
*Highest Level of Education Completed*									
No School		**6**	27%		3	14%		3	14%
Primary		**7**	32%		5	23%		2	9%
Middle		**5**	23%		3	14%		2	9%
Secondary		**3**	14%		0	0%		3	14%
Post-Secondary		**0**	0%		0	0%		0	0%
Missing		**1**	5%		1	5%		0	0%
*Occupation*								
Current miner		**13**	59%		4	18%		9	41%
Non-miner		**9**	41%		7	32%		2	9%
Missing		0	0%		0	0%		0	0

### 3.2. Perceived Stress

On the PSS items, the majority of respondents were “sometimes” upset, unable to control important things in life, and nervous or stressed (data not shown). More women than men responded “never” to those items. Compared to men, a greater percentage of women than men were “never” (17% *vs*. 10%) or “very often” (40% *vs*. 11%) nervous or stressed), but this difference was not significant. As Cronbach’s α was −0.978 for the five PSS items, they were not combined to create a total PSS score.

### 3.3. Salivary Cortisol

Intraassay coefficients of variation for salivary cortisol samples were 0–16.55%, with the majority (93%) being under 10%. One evening sample over three standard deviations higher than all other values was excluded as an outlier. Evening and morning samples were discarded for one participant who reported taking both samples at the same time. The mean cortisol level, across participants with three valid samples, was 3.55 ± 1.19 nmol/L (n = 17) and mean morning to evening change across participants with valid evening and morning samples was −1.44 ± 4.27 nmol/L (n = 18), indicating a decline in cortisol from morning to evening.

**Figure 1:**
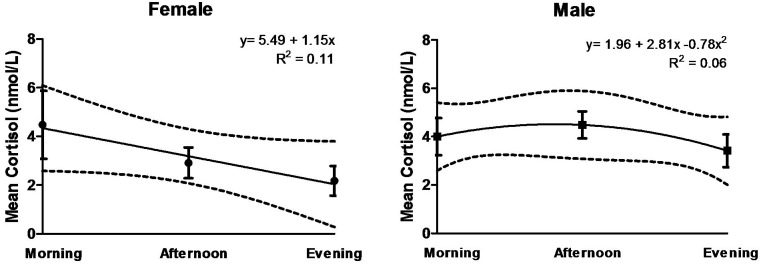
Change in salivary cortisol measurements over sampling period (morning to evening) for females (n = 9) and males (n = 8). Total duration between afternoon and morning samples ranged from 13.4–19.4 hours.

Patterns in morning to evening change for women and men are shown in Figure 1. Compared to women, men showed less of a decline in cortisol from morning to evening (−2.30 ± 4.86 nmol/L and −0.59 ± 3.93, respectively). This difference, however, was not significant (*p* = 0.755). Morning to evening change was similar for miners and non-miners, and no trend was observed for level of education completed.

### 3.4. Heart Rate

**Figure 2:**
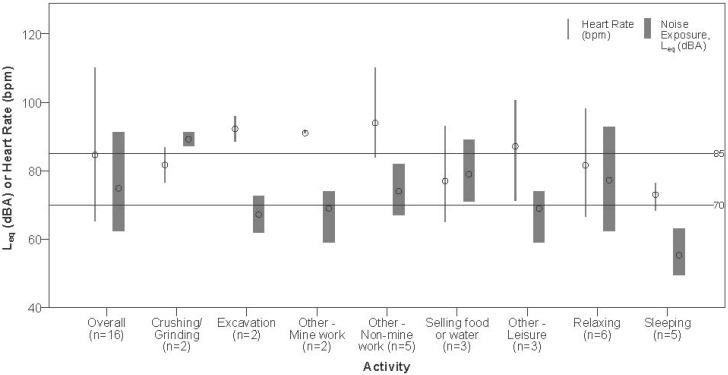
Paired noise and heart rate (HR) measurements. Bars represent range and dots represent means compared to the World Health Organization (WHO) 24-hour community noise exposure recommendation for hearing loss (70 dBA) and NIOSH 8-hour recommended occupational exposure limit (85 dBA). L_eq_ measurements differ from Table 2 because HR did not record continuously for most participants. “Other—Mine work” includes draining mine pit water and sifting/shanking. “Other—Non-mine work” includes plastering, seamstress work, selling drinks (bar), and retail vending from home. “Other—Leisure” activities include bathing, cooking, walking kids to school, shopping or market, and washing.

Across all 16 participants with valid measurements, a total of 5925 minutes of HR data was collected, with a subject mean duration of 370.3 ± 332.4 min, substantially less than the 1440 minutes in the nominal 24-hour monitored period. Per-person means for HR, overall and by activity, can be seen in Figure 2.

### 3.5. Noise

When asked to choose the biggest source of noise in their community, 50% of participants said ASGM activities were the biggest source while 37% chose the nearby Chinese-operated industrial gold mine. Thirty percent reported being not bothered by high noise at work, and 60% said they were bothered “a little”. Only 10% were bothered “a great deal.” A majority (70%) of people thought noise exposures at work were loud enough to harm their hearing, but only 45% believed the same about exposures outside of work. No participants were observed using hearing protection, and it was assumed that hearing protection was unavailable to the community.

Individual 24-hour average L_eq_ exposures ranged from 56.9 to 92.0 dBA, with a mean daily L_eq_ of 82.2 ± 7.3 dBA (mean monitoring duration 22.1 ± 1.9 hours). A single interval L_eq_ measurement that was determined to be an outlier (114.8 dBA) was excluded from analysis. Out of the 7775 total 3.75 minute L_eq_ values logged, 3184 (41.0%) were coded as 70/$\sqrt{2}$ to reflect the limit of detection (mean 159 ± 43, 3.75 minute intervals across participants, Supplementary Table 1). Twenty-one of 22 participants (95%) had an overall L_eq_ above the World Health Organization (WHO) exposure guideline of 70 dBA over 24 hours [30]. Table 2 displays mean L_eq_ for specific activities. Some work exposures were higher than leisure time exposures, and mine work exposures exceeded those of non-mine work on average. Crushing and grinding had the highest noise exposures and sleeping had the lowest noise exposures. No significant differences between men and women or miners and non-miners were found in daily L_eq_, leisure L_eq_, work L_eq_, or sleeping L_eq_. There was also no significant difference in work L_eq_ between people who thought noise exposure at work was loud enough to harm their hearing and those who thought otherwise. Interestingly, work L_eq_ was significantly lower (*p* = 0.04) for people who reported being bothered either “a little” or “a great deal” by noise at work (84.3 dBA, n = 12) compared to those who reported not being bothered at all (90.0 dBA, n = 5).

**Table 2:** Summary of personal noise exposures over sampling period.

		Total			Female			Male			Miner			Non-miner			Dosimetry measurement	
		Leq (dBA)			Leq (dBA)			Leq (dBA)			Leq (dBA)			Leq (dBA)			Duration (hr)	
	*N*	*Mean*	*SD*		*Mean*	*SD*		*Mean*	*SD*		*Mean*	*SD*		*Mean*	*SD*		*Mean*	*SD*
**Daily**	**22**	**82.2**	**7.3**		**82.2**	**4.4**		**82.3**	**10.0**		**82.8**	**8.8**		**81.5**	**4.6**		22.2	1.9
*Activity*																		
Leisure	17	81.9 *	8.2		83.0	3.8		80.9	11.0		81.7	9.6		82.2	4.2		6.0	5.0
Work	19	86.1 *	5.2		85.2	5.5		87.3	5.6		87.6	5.2		83.9	5.5		10.3	5.0
Sleeping	21	65.0	11.1		61.1	8.3		69.3	12.5		68.7	11.6		58.9	7.2		7.9	2.3
Non-mine work	13	80.1	16.3		85.2	5.8		63.4	30.1		74.1	25.9		83.9	5.5		9.4	5.2
Mine work	7	89.4	3.6		87.8	4.2		90.1	3.6		89.4	3.6		-	-		8.8	4.9
Grinding or crushing	3	92.4	2.0		-	-		92.4	2.0		92.4	2.0		-	-		10.1	7.1
Sifting or shanking	2	89.0	2.5		89.0	2.5		-	-		89.0	2.5		-	-		3.9	2.2
Excavation	3	84.2	3.0		-	-		84.2	3.0		84.2	3.0		-	-		8.4	3.4

* Significant difference (*p* < 0.05) between mean Leisure and Work L_eq_ based on Wilcoxon Signed Rank test.

**Table 3:** Linear regression model of changes in cortisol level (nmol/L).

Variable	Unstandardized Coefficients	95% CI
Intercept	−16.8	−32.6–1.02
Age (Years)	−0.06	−0.15–0.02
Sex (Male)	−0.51	−2.37–1.35
Smoker (Yes)	0.71	−2.21–3.63
Times Between Cortisol Samples (Hours)	−0.34	−0.76–0.09
L_eq_ (dBA)	0.25	0.08–0.42

Dependent variable: ΔCortisol_afternoon-evening_.

### 3.6. Salivary Cortisol and Noise

The afternoon to evening change in cortisol was positively correlated with corresponding L_eq_ measurements, with Spearman’s rho of 0.683 (*p* = 0.004, n = 17), meaning higher noise levels were associated with an increase in cortisol throughout the day. Adjusting for age, sex, smoking status, and time between samples in linear regression, a 1 dBA increase in L_eq_ was associated with 0.25 nmol/L increase in cortisol between morning and evening (95% CI: 0.08, 0.42, Adj R^2^ = 0.502, n = 17) (Table 3).

### 3.7. Heart Rate and Noise

Mean HR was not significantly correlated with morning to evening change in cortisol or the corresponding mean L_eq_ over the sampling period; however, variation in HR over time, as measured by the standard deviation of HR, was highly correlated with variation in L_eq_ for some individual participants, and the SD of the mean HR showed a moderate positive correlation with the SD of daily L_eq_ (Spearman’s rho = 0.532, *p* = 0.03). A mixed linear regression model for repeated measurements showed that HR (with a 3.75 minute interval between each measurement) was significantly associated with noise over sampling period, after adjusting for age and sex. With each 1 bpm increase in HR, L_eq_ increased 0.29 dBA (95% CI: 0.26–0.32, *p* < 0.001) (Table 4). Similarly, after controlling for sex and age in a backwards stepwise regression with HR standard deviation as the outcome, a 1 dBA increase in L_eq_ SD was associated with a 0.5 bpm increase in HR SD (95% CI: 0.04–0.9, Adj. R^2^ = 0.229, n = 16) (Table 5).

**Table 4:** Mixed effect linear regression model of the mean of heart rate (bpm) for a 3.75 minute interval.

Variable	Unstandardized Coefficients	95% CI
Intercept	57.5	37.2–77.7
Age (Years)	0.24	−0.31–0.78
Sex (Male)	5.37	−16.5–6.84
L_eq_ (dBA)	0.29	0.26–0.32

Dependent variable: HR_mean

**Table 5:** Linear regression model of the standard deviation of heart rate (bpm) over entire monitoring duration.

Variable	Unstandardized Coefficients	95% CI
Intercept	3.1	−5.4–11.8
Age (Years)	0.5	−0.2–0.1
Sex (Male)	1.1	−2.5–4.7
L_eq_ (dBA)	0.5	0.04–0.9

Dependent variable: HR_SD.

## 4. Discussion

Health vulnerabilities in ASGM communities arise both indirectly (through social structures and migration) and directly (through mining activities that introduce noise and other environmental hazards). Documentation of exposures to stressors in these communities can lead to a better understanding of how they cumulatively influence well-being and ultimately help identify appropriate interventions. By exploring the relationship between stress and noise, this study begins to elucidate the interaction between a few of these indirect and direct vulnerabilities. In terms of noise exposure, 95% of subjects in the 2013 cross-sectional study were over the WHO recommended guideline of 70 dBA over 24 hours, suggesting these individuals are at risk for hearing loss and perhaps other health outcomes [30]. High noise levels were ubiquitous, with daily mean L_eq_ exposures around 80 dBA for both genders and for both miners and non-miners. Adverse changes in salivary cortisol levels were associated with increasing daily noise exposure. This study also documented noise levels and changes in heart rate associated with common small-scale mining and other occupational activities. A crude measure of heart rate variation (SD of heart rate) increased with variation in noise level (SD of L_eq_). A mixed model also indicated an association between and noise exposure.

### 4.1. Noise

Workplace noise exposure is of particular concern for miners, and the outcome of excessive noise exposures, noise-induced hearing loss (NIHL), is unfortunately common. For example, in the African subregion that includes Ghana, 25% of hearing loss in males and 11% in females is attributed to noise [31]. In a Ghanaian surface gold mine, 23% of workers showed signs of NIHL [32]. While not all of this hearing loss may be attributable to work in the mine, noise levels above 85 dBA, the National Institute for Occupational Safety and Health (NIOSH) Recommended Exposure Limit over an 8 hour period [33], were recorded at four out of five areas of the mine that were surveyed [34]. In a study of Nicaraguan gold miners, audiometric tests showed hearing impairment in 35% of subjects (21 of 59 participants). These results, however, did not correlate with estimated noise exposure, though it should be noted that noise exposure was crudely determined by self-reported time spent doing various activities [35].

While the 8-hour 85 dBA recommended occupational exposure limit is pertinent to ASGM workers, our results suggest that all ASGM community members may be at risk for noise-induced hearing loss, as 95% of participants recorded noise exposures over the WHO recommended 24-hour guideline of 70 dBA [36]. In addition to hearing loss, workers and the public in ASGM communities may be at risk for other non-auditory effects of noise, including potentially deadly effects such as myocardial infarction [37]. Children also appear to be especially vulnerable to the effects of chronic noise exposure [38], with higher noise annoyance and lower reading comprehension in children attending schools with elevated noise levels [39]. Evidence for physiological effects carrying over into adulthood has not yet been gathered [40], but long-term consequences of learning disruptions raises serious concerns for the long-term success and well-being of children in these settings.

Noise exposure has been identified as a risk for miners [10,39], but actual noise levels have not been well documented in ASGM communities. This study suggests that not only miners but also community members could be at risk for noise-induced hearing loss because of noise exposure. Our observation that L_eq_ was significantly lower for people who reported being bothered by noise at work to those who reported not being bothered could be an indication of hearing loss. Observations and reports by participants that mining and other noise sources are spread throughout the community and are not limited to standard work hours during the day. While every ASGM community is different, many feature intermingling of mining activities and community life. Documenting noise in this small ASGM community provides a baseline measurement and approach to monitoring noise that can inform practice and policy in the industry moving forward.

### 4.2. Stress

Reference ranges for salivary cortisol vary depending on populations, sampling, and analysis techniques. Mean morning and evening cortisol levels here were slightly lower than those reported in other studies [40,41,42,43]; however, our methodology did not capture the morning rise in cortisol expected after awakening. Changes in physiological stress (as assessed by salivary cortisol) here were consistent with patterns associated with chronic stress [17,41,44], as mean cortisol levels were only slightly lower in the evening than in the morning. When paired with noise exposure, these results suggest that increased noise may cause an increase in the biochemical stress response throughout the day. This finding is consistent with some of the existing literature [24,43,44,45,46], but not all studies have found such an association [39,47]. There are myriad physical and psychosocial factors that can influence cortisol levels [48]. Confounders to daily cortisol patterns include age, gender, menstrual cycle, hormonal status, alcohol consumption, smoking, and certain medications [27], only some of which were controlled for in this study. Nevertheless, our finding that variation in standard deviation of HR and standard deviation of L_eq_ provides further evidence of a measurable physiological stress response to noise.

### 4.3. Limitations

With a small sample size, these results should not be taken as representative of all ASGM communities, especially given the diversity that exists in the sector. Additionally, the lack of repeated sampling with salivary cortisol and noise exposure should be considered. Due to time and logistical constraints, we collected saliva samples in the afternoon, evening, and morning, respectively, and conducted noise monitoring between the afternoon of the survey and the following morning. Because of this, and because of concerns about the quality of the morning cortisol samples, we were not able to evaluate morning to afternoon or morning to evening changes in cortisol levels. Confounders in salivary cortisol measurement were controlled for as closely as possible in data collection and analysis, but the fact that evening samples preceded morning samples as well as our reliance on self-reporting for protocol adherence limits our findings. The high limit of noise detection in dosimeters (70 dBA) means that low-level noise exposures were not accurately characterized, and values below the limit of detection had to be inferred via a commonly-used statistical approach. Similarly, the heart rate monitors used did not directly measure heart rate variability, the preferred measure for evaluating the association between noise and stress, but rather average heart rate. Our use of a commercially-available, off-the-shelf heart rate monitor represented a necessary tradeoff between instrument durability, accuracy, and temporal resolution. The presence of recall bias in the activities reported by subjects is a real possibility; ideally, participants would have filled out the activity log on their own as they changed activities, but this was not possible due to the high prevalence of illiteracy among participants. Nonetheless, despite these limitations, our study is the first to make the association between increased cortisol and increased noise levels in a small-scale mining setting with both occupational and community noise exposures.

## 5. Conclusions

This study provides useful baseline data on two understudied hazards—stress and noise—in ASGM communities. Measured 24-hour noise exposures were consistently above the WHO guideline of 70 dBA, and were associated with increases in both biochemical and physiological measures of stress. If noise levels interfere with educational attainment or add additional psychosocial or physiological stress in these mining communities, residents may be at an even greater disadvantage for long-term well-being. While small-scale mining communities are diverse in terms of layout, activities, and exposures, documenting stress and noise in one of these communities gives a snapshot into conditions that could be faced by the estimated 15 million people worldwide who work in the industry and the 100 million people on the periphery of ASGM economies [49].

Research on health in ASGM communities has often focused on the unique chemical exposures [1,2,3] and social dynamics associated with these activities [6,10,50,51], but few previous studies have integrated both the physical and social vulnerabilities potentially faced by ASGM community residents as we have done here. Further research is needed to document stress and noise exposure conditions in similar communities, to more fully characterize health determinants in mining communities, and to assess actual health impacts associated with these exposures. The results of such research can inform policies and practices to begin immediately addressing these issues in similar vulnerable communities.

## Supplementary Files

Supplementary File 1
