# Supplementary material for: The Association between Noise, Cortisol and Heart Rate in a Small-Scale Gold Mining Community—A Pilot Study
Source: Int J Environ Res Public Health. 2015 Aug 21;12(8):9952–66. doi: 10.3390/ijerph120809952 (PMC4555322; doi:10.3390/ijerph120809952)
Supplement: Supplementary File 1 [file ijerph-12-09952-s001.pdf]

Supplementary

The Association between Noise, Cortisol and Heart Rate in a Small-Scale Gold Mining Community—A Pilot Study

Table S1. Summary of the valid (*i.e.*, >0 dBA) L_{EQ} noise measurement intervals over sampling period by subject and activity (N = 7775 monitored 3.75 minute intervals); 0 dBA intervals reassigned as 70 dBA.

**Leq**	**Valid Data %**		**Missing Data**
	**Mean (%)**	**SD %**	***% mean***
**Daily**	59.0	12.8	46.6
Activity			
Leisure	72.4	24.7	27.6
Work	80.0	17.6	20.0
Sleeping	16.0	14.1	84.0
Non-mine work	75.9	26.5	24.1
Mine work	79.2	17.2	20.8
Grinding or crushing	89.5	13.9	10.5
Sifting or shanking	94.8	7.3	5.2
Excavation	55.2	8.9	44.8

© 2015 by the authors; licensee MDPI, Basel, Switzerland. This article is an open access article distributed under the terms and conditions of the Creative Commons Attribution license (http://creativecommons.org/licenses/by/4.0/).
